# Adverse Pregnancy Outcomes and Long-term Maternal Kidney Disease: A Systematic Review and Meta-analysis

**DOI:** 10.1001/jamanetworkopen.2019.20964

**Published:** 2020-02-12

**Authors:** Peter M. Barrett, Fergus P. McCarthy, Karolina Kublickiene, Sarah Cormican, Conor Judge, Marie Evans, Marius Kublickas, Ivan J. Perry, Peter Stenvinkel, Ali S. Khashan

**Affiliations:** 1 School of Public Health, University College Cork, Cork, Ireland; 2 Irish Centre for Maternal & Child Health, University College Cork, Cork, Ireland; 3 Department of Obstetrics & Gynaecology, Cork University Maternity Hospital, Cork, Ireland; 4 Division of Renal Medicine, Department of Clinical Intervention, Science and Technology, Karolinska Institutet, Stockholm, Sweden; 5 Department of Nephrology, University Hospital Galway, Galway, Ireland; 6 Department of Obstetrics & Gynaecology, Karolinska University Hospital, Stockholm, Sweden

## Abstract

**Importance:**

Adverse pregnancy outcomes, such as hypertensive disorders of pregnancy, gestational diabetes, and preterm delivery, are associated with increased risk of maternal cardiovascular disease. Little is known about whether adverse pregnancy outcomes are associated with increased risk of maternal chronic kidney disease (CKD) and end-stage kidney disease (ESKD).

**Objective:**

To review and synthesize the published literature on adverse pregnancy outcomes (hypertensive disorders of pregnancy, gestational diabetes, and preterm delivery) and subsequent maternal CKD and ESKD.

**Data Sources:**

PubMed, Embase, and Web of Science were searched from inception to July 31, 2019, for cohort and case-control studies of adverse pregnancy outcomes and maternal CKD and ESKD.

**Study Selection:**

Selected studies included the following: a population of pregnant women, exposure to an adverse pregnancy outcome of interest, and at least 1 primary outcome (CKD or ESKD) or secondary outcome (hospitalization or death due to kidney disease). Adverse pregnancy outcomes included exposure to hypertensive disorders of pregnancy (preeclampsia, gestational hypertension, or chronic hypertension), preterm delivery (<37 weeks), and gestational diabetes. Three reviewers were involved in study selection. Of 5656 studies retrieved, 23 were eligible for inclusion.

**Data Extraction and Synthesis:**

The Meta-analyses of Observational Studies in Epidemiology (MOOSE) guidelines were followed throughout. Three reviewers extracted data and appraised study quality. Random-effects meta-analyses were used to calculate overall pooled estimates using the generic inverse variance method.

**Main Outcomes and Measures:**

Primary outcomes included CKD and ESKD diagnosis, defined using established clinical criteria (estimated glomerular filtration rate or albuminuria values) or hospital records. The protocol for this systematic review was registered on PROSPERO (CRD42018110891).

**Results:**

Of 23 studies included (5 769 891 participants), 5 studies reported effect estimates for more than 1 adverse pregnancy outcome. Preeclampsia was associated with significantly increased risk of CKD (pooled adjusted risk ratio [aRR], 2.11; 95% CI, 1.72-2.59), ESKD (aRR, 4.90; 95% CI, 3.56-6.74), and kidney-related hospitalization (aRR, 2.65; 95% CI, 1.03-6.77). Gestational hypertension was associated with increased risk of CKD (aRR, 1.49; 95% CI, 1.11-2.01) and ESKD (aRR, 3.64; 95% CI, 2.34-5.66). Preterm preeclampsia was associated with increased risk of ESKD (aRR, 5.66; 95% CI, 3.06-10.48); this association with ESKD persisted for women who had preterm deliveries without preeclampsia (aRR, 2.09; 95% CI, 1.64-2.66). Gestational diabetes was associated with increased risk of CKD among black women (aRR, 1.78; 95% CI, 1.18-2.70), but not white women (aRR, 0.81; 95% CI, 0.58-1.13).

**Conclusions and Relevance:**

In this meta-analysis, exposure to adverse pregnancy outcomes, including hypertensive disorders of pregnancy, gestational diabetes, and preterm delivery, was associated with higher risk of long-term kidney disease. The risk of ESKD was highest among women who experienced preeclampsia. A systematic approach may be warranted to identify women at increased risk of kidney disease, particularly after hypertensive disorders of pregnancy, and to optimize their long-term follow-up.

## Key Points

**Question:**

Are adverse pregnancy outcomes associated with risk of long-term kidney disease in women?

**Findings:**

In this systematic review and meta-analysis of 23 studies (5 769 891 participants), the risk of end-stage kidney disease was 4.9 times higher in women who had preeclampsia, and 3.6 times higher in women who had gestational hypertension vs women with normotensive pregnancies. The risk of end-stage kidney disease was 2.1 times higher in women who had preterm deliveries compared with women who delivered at term.

**Meaning:**

Women who experience hypertensive disorders of pregnancy and other adverse pregnancy outcomes may warrant closer surveillance for long-term kidney disease.

## Teaser

This systematic review and meta-analysis examines the published literature on adverse pregnancy outcomes (hypertensive disorders of pregnancy, gestational diabetes, and preterm delivery) and subsequent maternal chronic kidney disease and end-stage kidney disease.

## Introduction

Pregnancy is increasingly regarded as a metabolic stress test, which may unmask underlying vascular disease and endothelial dysfunction.^1^ The risk of long-term cardiovascular disease is increased among women with a history of preeclampsia, gestational hypertension, or gestational diabetes (GDM).^2,3,4,5^ Comparatively little is known about the long-term risk of chronic kidney disease (CKD) and end-stage kidney disease (ESKD) in women who have experienced adverse pregnancy outcomes. It is plausible that women who experience hypertensive disorders of pregnancy (HDP) have a higher risk of long-term kidney disease than women with normotensive pregnancies. Women exposed to preeclampsia have an increased risk of microalbuminuria,^6,7^ and longitudinal studies have suggested that they may be at increased risk of long-term kidney disease.^8,9^ It is uncertain whether gestational hypertension and chronic hypertension may have similar associations with the risk of maternal kidney disease.

Preterm delivery and delivery of a growth-restricted or low-birth-weight infant have been identified as risk factors for maternal cardiovascular disease,^10,11,12,13^ but it is unclear whether they are independently associated with increased risk of future kidney disease. It is possible that these factors mediate the association between HDP or other pregnancy-related disorders and CKD or ESKD.

Women who develop GDM are predisposed to lasting vascular endothelial dysfunction.^14,15^ It is plausible that this dysfunction may in itself increase their risk of kidney disease, even if they never develop type 2 diabetes.^1^ By 9 to 16 years post partum, women exposed to GDM may experience early renal damage,^16^ but it is uncertain whether they are at risk of clinically significant CKD.

This systematic review and meta-analysis aims to synthesize the available evidence on the associations between adverse pregnancy outcomes and long-term maternal CKD and ESKD.

## Methods

### Data Sources and Search Strategy

A systematic search of PubMed, Embase, and Web of Science was undertaken from inception of the databases until July 31, 2019. We sought studies with the following: a population of pregnant women, exposure to an adverse pregnancy outcome of interest, a comparison group of women with no corresponding adverse pregnancy outcome, and at least 1 primary outcome (CKD or ESKD) or secondary outcome (hospitalization or death due to kidney disease). This study followed the Meta-analysis Of Observational Studies in Epidemiology (MOOSE) reporting guidelines.^17^

The adverse pregnancy outcomes included exposure to HDP, specifically, preeclampsia, gestational hypertension, chronic hypertension, or preeclampsia superimposed on chronic hypertension (PSH), preterm delivery (<37 weeks), delivery of an infant with low birth weight (including small for gestational age and intrauterine growth restriction), and GDM. Each exposure could be defined either using established clinical criteria, hospital records, or self-reporting of a physician’s diagnosis. For CKD and ESKD, they could be defined using established clinical criteria (ie, estimated glomerular filtration rate or albuminuria values) or hospital records. The protocol for this systematic review was registered on PROSPERO (CRD42018110891) and subsequently published.^18^

### Study Selection

**Figure 1.**
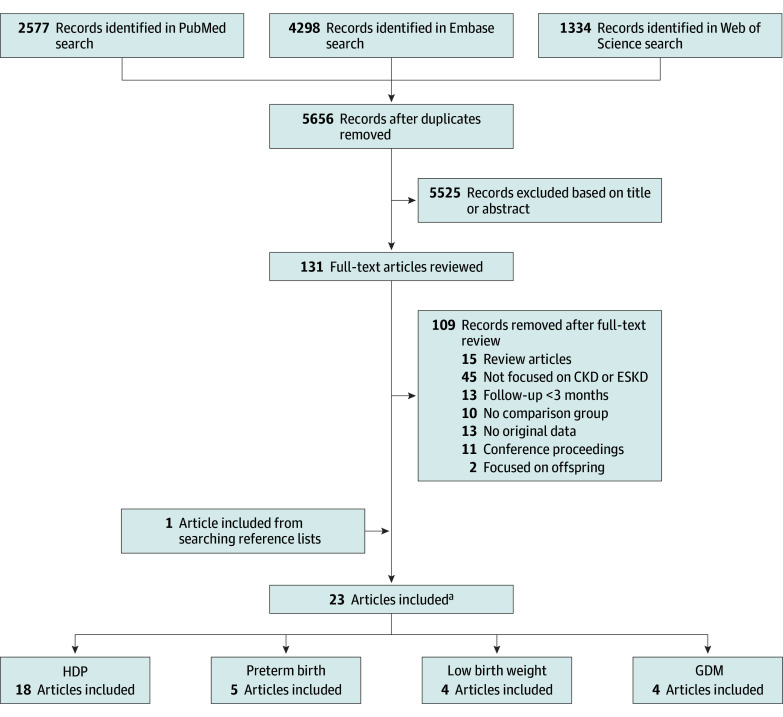
Flow Diagram of Studies Selected for Inclusion in Systematic Review CKD, indicates chronic kidney disease; ESKD, end-stage kidney disease; GDM, gestational diabetes mellitus; and HDP, hypertensive disorders of pregnancy. ^a^Five of the included studies contained effect estimates for more than 1 relevant adverse pregnancy outcome.

The process of study selection is outlined in Figure 1. Two of us (P.M.B. and S.C.) independently reviewed titles and abstracts of all studies. Peer-reviewed English-language case-control and cohort studies (prospective or retrospective) were included if they published original effect estimates of the association between at least 1 adverse pregnancy outcome of interest and a primary or secondary outcome.

Studies were excluded if maternal renal outcomes were ascertained less than 3 months post partum to avoid misclassification with pregnancy-related acute kidney injury. Studies were also excluded if they were restricted to women with prepregnancy kidney disease, if they used changes in biological or laboratory markers of kidney function as the only outcome (eg, change in estimated glomerular filtration rate or microalbuminuria) without information about CKD or ESKD diagnosis, and if they focused on renal outcomes among offspring instead of mothers.

### Data Extraction and Quality Appraisal

Two investigators (P.M.B. and C.J.) extracted data independently from eligible studies. Where information was not directly available from the studies, authors were contacted to request this information, and at least 1 follow-up reminder email was sent. The bias classification tool of McDonald et al^19^ was used to assess 6 types of bias commonly associated with observational studies (selection, exposure, outcome, attrition, analytic, and confounding). For every study, the presence of each type of bias was classified as minimal, low, moderate, high, or not reported, and an overall likelihood of bias score was measured.

### Statistical Analysis

Data were analyzed using Review Manager software, version 5.3.^20^ Random-effects meta-analyses were used to calculate overall pooled estimates of the associations between adverse pregnancy outcomes and subsequent maternal CKD or ESKD. Both crude and adjusted effect estimates were displayed using the generic inverse variance method. Post hoc analyses were also conducted using the Sidik-Jonkman method in Stata, version 15.0 (Stata Corp), given the small number of included studies in each meta-analysis.^21^

Forest plots were used to display pooled estimates, with corresponding 95% CIs. Statistical heterogeneity was explored based on *I*^2^ values and τ^2^ statistics. In the meta-analyses, odds ratios, hazard ratios, and relative risks (RRs) were used as approximations of each other. Under the rare disease assumption, odds ratios and RRs are commonly used to approximate each other,^22,23^ and hazard ratios may be considered as an extension of this approximation when the outcome is uncommon.^23,24^

Separate subgroup analysis by study type, follow-up time, measurement of outcome variables (self-report vs laboratory measurements vs medical records), CKD staging, and study quality were planned, but were not conducted owing to the small number of included studies. Subgroup analysis by race/ethnicity of participating women (white vs black) was possible only in the case of GDM. We planned to assess publication bias using funnel plots, but a minimum of 10 unique studies should be included in order to do so.^25^ We had fewer studies in each separate meta-analysis, and thus could not assess publication bias using funnel plots.

We presented adjusted effect estimates based on the definitions outlined in each individual study. However, we also conducted sensitivity analyses whereby meta-analyses were restricted to studies reporting effect estimates adjusted for important maternal comorbidities (CKD, hypertension, and diabetes).

We used the formula of Levin^26^ to calculate the population-attributable fraction of preeclampsia on CKD and ESKD: *P* × (RR − 1)/*P* × (RR − 1) + 1, where *P* is the pooled prevalence of exposure in all cohort studies included in a given meta-analysis, and RR is the pooled adjusted RR from the meta-analysis.

## Results

The search yielded 5656 unique results. Of these, 23 articles were included in the systematic review, covering 14 unique study populations (5 769 891 participants). Five articles provided effect estimates for more than 1 adverse pregnancy outcome of interest.

The sample size of included studies ranged widely, from 132 participants in an American case-control study^27^ to a registry-based study of 1 598 043 Canadian women.^28^ Ten studies were conducted in Europe,^8,9,29,30,31,32,33,34,35,36^ 7 in Asia,^37,38,39,40,41,42,43^ 4 in North America,^27,28,44,45^ and 2 in Australia.^46,47^ Three studies had moderate risk of bias,^34,39,45^ but the remaining 20 studies were assessed as having minimal or low risk of bias.

The summary results of all meta-analyses are presented in Table 1.^8,9,27,28,29,30,31,32,33,34,35,36,37,38,39,40,41,42,43,44,45,46,47^ Detailed characteristics of the 18 articles that provided effect estimates for HDP and maternal kidney disease are presented in Table 2.^8,9,27,28,29,30,31,32,33,34,35,36,37,38,39,42,46,47,48^ The data relating to preterm birth are presented in eTable 1 in the Supplement, data relating to delivery of low-birth-weight (or small for gestational age) infants are presented in eTable 2 in the Supplement, and data relating to GDM are presented in eTable 3 in the Supplement. Nine studies provided effect estimates for any HDP and ESKD.^8,27,28,29,30,31,37,38^ Four of these studies were excluded from the meta-analysis, either owing to overlap of study populations or because they were restricted to women with higher baseline risk of ESKD.^29,30,31,38^ All the remaining studies provided adjusted effect estimates for preeclampsia and ESKD, of which 2 also provided adjusted estimates for gestational hypertension, chronic hypertension, and PSH in mutually exclusive groups.

**Table 1.** Summary Results of Meta-analyses^a^

Outcome	No. of Studies	Reference No.	No. of Participants	No. of Outcomes	Pooled RR (95% CI)	*I*^2^, %	τ^2^
**Preeclampsia**							
ESKD							
Crude	5	8, 27, 28, 35, 37	4 479 523	1737	6.16 (4.42-8.57)	79	0.10
Adjusted (any)	5	8, 27, 28, 35, 37	4 479 523	1737	4.90 (3.56-6.74)	73	0.09
Adjusted for comorbidities	5	8, 27, 28, 35, 37	4 479 523	1737	4.90 (3.56-6.74)	73	0.09
CKD							
Crude	3	9, 32, 33	1 097 495	4699	2.27 (1.48-3.49)	80	0.09
Adjusted (any)	3	9, 32, 33	1 097 495	4699	2.11 (1.72-2.59)	35	0.01
Adjusted for comorbidities	1	9	1 072 330	3901	2.27 (2.02-2.55)	NA	NA
Kidney-related hospitalization							
Crude	2	36, 42	131 224	468	1.79 (0.71-4.51)	92	0.41
Adjusted (any)	3	36, 42, 47	162 880	1051	2.65 (1.03-6.77)	92	0.62
Adjusted for comorbidities	0	NA	NA	NA	NA	NA	NA
**Gestational Hypertension**							
ESKD							
Crude	2	28, 37	2 542 517	806	4.37 (1.74-10.98)	67	0.31
Adjusted (any)	2	28, 37	2 542 517	806	3.64 (2.34-5.66)	7	0.01
Adjusted for comorbidities	2	28, 37	2 542 517	806	3.64 (2.34-5.66)	7	0.01
CKD							
Crude	2	32, 33	25 165	798	2.56 (1.09-2.22)	55	0.04
Adjusted (any)	2	32, 33	25 165	798	1.49 (1.11-2.01)	40	0.02
Adjusted for comorbidities	0	NA	NA	NA	NA	NA	NA
Kidney-related hospitalization							
Crude	2	32, 36	49 705	1010	1.04 (0.92-1.17)	0	0.00
Adjusted (any)	2	36, 47	66 510	939	1.84 (0.60-5.67)	90	0.60
Adjusted for comorbidities	0	NA	NA	NA	NA	NA	NA
**Chronic Hypertension**							
ESKD							
Crude	2	28, 37	2 542 517	806	16.87 (11.31-25.15)	0	0.00
Adjusted (any)	1	37	944 474	258	15.99 (5.89-43.41)	NA	NA
Adjusted for comorbidities	1	37	944 474	258	15.99 (5.89-43.41)	NA	NA
CKD							
Crude	1	33	10 314	144	1.62 (0.88-3.00)	NA	NA
Adjusted (any)	1	33	10 314	144	1.23 (0.67-2.26)	NA	NA
Adjusted for comorbidities	0	NA	NA	NA	NA	NA	NA
**Superimposed Preeclampsia**							
ESKD							
Crude	2	28, 37	2 542 517	806	48.97 (26.09-91.93)	41	0.09
Adjusted (any)	1	37	944 474	258	44.72 (22.59-88.52)	NA	NA
Adjusted for comorbidities	1	37	944 474	258	44.72 (22.59-88.52)	NA	NA
CKD							
Crude	1	33	10 314	144	1.56 (0.38-6.42)	NA	NA
Adjusted (any)	1	33	10 314	144	1.24 (0.28-5.49)	NA	NA
Adjusted for comorbidities	0	NA	NA	NA	NA	NA	NA
**Preterm Delivery (no preeclampsia)**							
ESKD							
Crude	3	8, 28, 29	2 169 957	1073	3.21 (2.35-4.39)	57	0.04
Adjusted (any)	3	8, 28, 29	2 169 957	1073	2.09 (1.64-2.66)	18	0.01
Adjusted for comorbidities	3	8, 28, 29	2 169 957	1073	2.09 (1.64-2.66)	18	0.01
CKD							
Crude	0	NA	NA	NA	NA	NA	NA
Adjusted (any)	0	NA	NA	NA	NA	NA	NA
Kidney-related hospitalization							
Crude	1	43	99 338	132	2.90 (2.00-4.20)	NA	NA
Adjusted (any)	1	43	99 338	132	2.70 (1.80-3.90)	NA	NA
Adjusted for comorbidities	0	NA	NA	NA	NA	NA	NA
**Preterm Preeclampsia**							
ESKD							
Crude	3	8, 29, 35	1 938 355	935	7.66 (3.16-18.55)	86	0.52
Adjusted (any)	3	8, 29, 35	1 938 355	935	5.66 (3.06-10.48)	59	0.17
Adjusted for comorbidities	3	8, 29, 35	1 938 355	935	5.66 (3.06-10.48)	59	0.17
CKD							
Crude	0	NA	NA	NA	NA	NA	NA
Adjusted (any)	1	9	1 072 330	3901	3.93 (2.90-5.33)	NA	NA
Adjusted for comorbidities	1	9	1 072 330	3901	3.93 (2.90-5.33)	NA	NA
**Gestational Diabetes**							
ESKD							
Crude	0	NA	NA	NA	NA	NA	NA
Adjusted (any)	0	NA	NA	NA	NA	NA	NA
CKD							
Crude	3	40, 44, 45	136 504	6345	0.99 (0.66-1.49)	46	0.06
Adjusted (any)	2	44, 45	38 536	6231	1.04 (0.76-1.41)	21	0.01
Adjusted for comorbidities	2	44, 45	38 536	6231	1.04 (0.76-1.41)	21	0.01
Kidney-related hospitalization							
Crude	0	NA	NA	NA	NA	NA	NA
Adjusted (any)	1	42	96 370	112	1.90 (1.10-3.20)	NA	NA
Adjusted for comorbidities	0	NA	NA	NA	NA	NA	NA

Abbreviations: CKD, chronic kidney disease; ESKD, end-stage kidney disease; NA, not applicable; RR, risk ratio.

^a^ Meta-analysis was based on the generic inverse variance (DerSimonian-Laird) method. Each study’s sample RR was entered in RevMan, version 5.3, and the log (RR) was calculated. Then, based on the study’s reported 95% CIs (for the sample RR), RevMan calculated the SE. RevMan uses information from the log (RR) and either the lower or upper 95% CI to derive the SE and the value of the corresponding 95% CI.

**Table 2.** Characteristics of Studies Which Investigate Hypertensive Disorders of Pregnancy and Subsequent Maternal Kidney-Related Disease

Source	Country, Follow-up, y	Study Design, Data Source^a^	Sample Size, No.	Exposures	Outcomes, Measure of Effect	Exclusions	Confounders Adjusted	RR (95% CI)	
								Crude	Adjusted
Dai et al,^28^ 2018	Canada, median 15	RC, hospital records	1 598 043	HDP overall, GH, PE, CH, PE superimposed on CH, and nonspecific hypertension	ESKD hospitalization, HR	Maternal age <15 or >44 y, multiple gestations, previous kidney disease, DM, GDM, SLE, HUS, thrombotic microangiopathy, and hypertension secondary to kidney disease	Maternal age, region, time period, obesity, preterm delivery, intrauterine death, fetal distress, placental disorders or abruption, oligohydramnios, prolonged pregnancy, postpartum hemorrhage, DVT, cardiac disease, blood transfusion, and cesarean delivery	HDP overall, 5.8 (4.8-7.0); GH, 3.0 (1.9-4.7); PE, 6.0 (4.7-7.6); CH, 15.6 (10.1-24.2); PE superimposed on CH, 35.2 (17.5-70.8); nonspecific, 5.0 (3.0-8.4)	GH, 3.27 (2.10-5.09); PE, 4.67 (3.63-5.99); nonspecific 4.60 (2.75-7.71)
Kattah et al,^27^ 2017	United States, median 18	CC, US Renal Data System	132	HDP overall, PE	ESKD, OR	Previous ESKD	Obesity, race/ethnicity, education, prepregnancy hypertension, and DM	HDP overall, 3.34 (1.32-8.47); PE, 4.0 (1.21-13.28)	PE, 3.25 (0.93-11.37) [adjusted only for obesity]
Sandvik et al,^29^ 2010	Norway, ≤37	RC, Norwegian Renal Registry	1481	PE	ESKD, RR	Multiple deliveries, previous kidney disease, and hypertension; all included women had preexisting diabetes	Year of birth, age, marital status, stillbirth, congenital malformations of offspring, and cesarean delivery in first pregnancy	PE, 1.3 (0.41-4.4); preterm PE, 2.8 (1.3-6.0)	PE, 1.3 (0.3-5.6); preterm PE, 2.9 (1.3-6.4)
Vikse et al,^8^ 2008	Norway, mean 27	RC, Norwegian Renal Registry	570 433	PE	ESKD, RR	Multiple pregnancies, previous kidney disease, hypertension, rheumatic disease, or DM	Year of delivery, maternal age, marital status, stillbirth, and congenital malformation of infant	4.7 (3.6-6.1)	3.2 (2.2-4.5)
Vikse et al,^30^ 2010	Norway, ≤16	RC, Norwegian Renal Registry	582	PE	ESKD, RR	None specified; study restricted to women who previously underwent kidney biopsy for suspected kidney damage	Age, eGFR, proteinuria, diastolic blood pressure, duration of kidney disease, interstitial fibrosis, and inflammation	1.2 (0.63-2.4)	1.1 (0.50-2.6)
Vikse et al,^31^ 2012	Norway, mean 20	RC, Norwegian Renal Registry	570 675	PE	ESKD, RR	Previous kidney disease, hypertension, rheumatic disease, or DM	Maternal age, marital status, number of siblings, and maternal education	PE without maternal sibling with PE, 5.95 (4.37-8.11); PE with maternal sibling with PE, 2.76 (0.88-8.63)	PE without maternal sibling with PE, 2.88 (1.69-4.90)
Wu et al,^37^ 2014	Taiwan, median 9	RC, National Health Insurance Research Database	944 474	HDP overall, PE, GH, CH, PE superimposed on CH	ESKD, HR	Previous kidney disease, DM, thrombotic microangiopathy, HUS, SLE, hypertension secondary to kidney disease, and women with HDP in >1 pregnancy	Maternal age, mode of delivery, number of deliveries, and complications (ie, early delivery, threatened labor, threatened premature labor)	HDP overall, 15.23 (11.07-20.95); GH, 7.85 (2.92-21.1); PE, 13.16 (8.69-19.92); CH, 25.02 (9.31-67.29); PE superimposed on CH, 66.95 (34.36-130.44)	HDP overall, 10.64 (7.53-15.05); GH, 5.82 (2.15-15.77); PE, 9.46 (6.10-14.68); CH, 15.99 (5.89-43.38); PE superimposed on CH, 44.72 (22.59-88.51)
Khashan et al,^35^ 2019	Sweden, median 7	RC, Swedish Renal Registry	1 366 441	PE	ESKD, HR	Multiple births, previous kidney disease, CVD, hypertension, or DM	Maternal age, BMI, education, country of origin, and smoking	Overall PE, 4.99 (3.93-6.33); preterm PE, 9.19 (5.16-16.35)	Overall PE, 4.96 (3.89-6.32); preterm PE, 8.76 (4.91-15.61)
Wang et al,^38^ 2013	Taiwan, mean 6	RC, National Health Insurance Research Database	240 048	HDP overall, PE, GH	CKD, ESKD, HR	Previous kidney disease, hypertension, SLE, or DM	Urban status, coronary artery disease, congestive heart failure, hyperlipidemia, hypertension, DM, and placental abruption	(1) For CKD: HDP overall, 10.8 (8.20-14.2); (2) for ESKD: HDP overall, 14.1 (9.76-20.3); PE, 15.9 (10.8-23.3); GH, 10.2 (5.89-17.6)	(1) For CKD: HDP overall, 9.38 (7.09-12.4) (2) for ESKD: HDP overall, 2.72 (1.76-4.22); PE, 3.19 (2.02-5.02); GH, 1.81 (0.99-3.30)
Kristensen et al,^9^ 2019	Denmark, mean 19	RC, multiple linked national registers	1 072 330	PE	CKD, HR	Age <15 y, previous kidney disease, CVD, autoimmune disease, hypertension, or DM	Maternal age, year, parity, GH, CH, gestational length, postpartum autoimmune disease, CVD, or DM	Term PE, 3.00 (2.67-3.37)	Term PE, 2.27 (2.02-2.55); early preterm PE, 3.93 (2.90-5.33); late preterm PE, 2.81 (2.13-3.71)
Männistö et al,^33^ 2013	Finland, mean 39	RC, multiple linked national registers	10 314	PE, GH	CKD, HR	Missing blood pressure data, death in first year post partum, multiple gestations, 2 completed pregnancies within same study year	Prepregnancy BMI, smoking, parity, DM, and socioeconomic status (age used as time scale)	PE, 0.74 (0.18-3.03); GH, 2.02 (1.25-3.26); PE superimposed on CH, 1.56 (0.38-6.42); CH, 1.62 (0.88-3.00)	PE, 0.75 (0.17-3.38); GH, 1.91 (1.18-3.09); PE superimposed on CH, 1.24 (0.28-5.44); CH, 1.23 (0.67-2.24)
Oishi et al,^39^ 2017	Japan, mean 31	RC, maternity health records	312	HDP	CKD, OR	Women with incomplete antenatal records or <5 blood pressure readings	Maternal age, BMI, hypertension, dyslipidemia, DM, and smoking	6.48 (1.60-26.30)	4.86 (1.04-22.62)
Paauw et al,^34^ 2018	Netherlands, median 11	PC, participants in PREVEND cohort study^48^	2782	HDP	CKD, HR	Women unaware of their history of pregnancy disorders	NA	1.04 (0.79-1.37)	Not reported
Ayansina et al,^32^ 2016	Scotland, 30-40	RC, multiple linked regional and national registers	14 851	PE, GH	CKD, kidney-related hospitalization, OR	Previous kidney disease, hypertension, multiple pregnancies, and temporary residents	Maternal age, BMI, socioeconomic status, and smoking	(1) For CKD: GH, 1.37 (1.15-1.64); PE, 2.02 (1.53-2.67); (2) for hospitalization: GH, 1.08 (0.84-1.40); PE, 1.42 (0.93-2.17)	(1) For CKD: GH, 1.36 (1.14-1.63); PE, 1.92 (1.45-2.56); (2) for hospitalization: GH, 1.02 (0.78-1.32); PE, 1.37 (0.90-2.10)
Kessous et al,^42^ 2015	Israel, mean 11	RC, clinical records at single institution	96 370	PE	Kidney-related hospitalization, HR	Previous CVD or kidney disease, congenital cardiac or kidney malformations, and multiple pregnancies	Maternal age, parity, obesity, DM, and smoking	2.93 (1.85-4.64)	3.7 (2.3-6.0)
Bhattacharya et al,^36^ 2012	Scotland	RC, multiple linked regional and national registers	34 854	PE, GH	Kidney-related hospitalization, kidney-related deaths, OR	Previous hypertension and multiple pregnancies	Year of birth, smoking status and social class at time of first pregnancy	(1) Hospitalization: PE, 1.14 (0.89-1.46); GH, 1.03 (0.90-1.18); (2) deaths: PE, 1.73 (0.52-5.81); GH, 1.71 (0.86-3.41)	(1) Hospitalization: PE, 1.20 (0.93-1.54); GH, 1.09 (0.95-1.25); (2) deaths: PE, 1.72 (0.51-5.79); GH, 1.81 (0.90-3.62)
Tooher et al,^47^ 2017	Australia, 20-29	RC, regional census of all hospital admissions	31 656	HDP in 4 groups: PE, GH, CH, and PE superimposed on CH	Kidney-related hospitalization, OR	Women whose HDP could not be categorized in one of the 4 groups	Maternal age, gestation at delivery, and parity	Not reported	Overall HDP, 2.76 (1.98-3.84); PE, 4.74 (2.19-10.20); GH, 3.45 (1.74-6.85)
Tooher et al,^46^ 2016	Australia, 20-29	RC, regional and national death registries	31 656	HDP in 4 groups: PE, GH, CH, and PE superimposed on CH	Kidney-related deaths, OR	Not reported	Maternal age, gestation at delivery, and parity	Overall HDP 1.72 (0.91-3.25)	Not reported

Abbreviations: BMI, body mass index; CC, case-control; CH, chronic hypertension; CKD, chronic kidney disease; CVD, cardiovascular disease; DM, diabetes mellitus; DVT, deep vein thrombosis; eGFR, estimated glomerular filtration rate; ESKD, end-stage kidney disease; GDM, gestational diabetes mellitus; GH, gestational hypertension; HDP, hypertensive disorders of pregnancy; HR, hazard ratio; HUS, hemolytic uremic syndrome; NA, not applicable; OR, odds ratio; PC, prospective cohort; PE, preeclampsia; PREVEND, Prevention of Renal and Vascular Endstage Disease; RC, retrospective cohort; RR, risk ratio; SLE, systemic lupus erythematosus.

^a^ Study design: CC, PC, or RC.

### Preeclampsia

**Figure 2.**
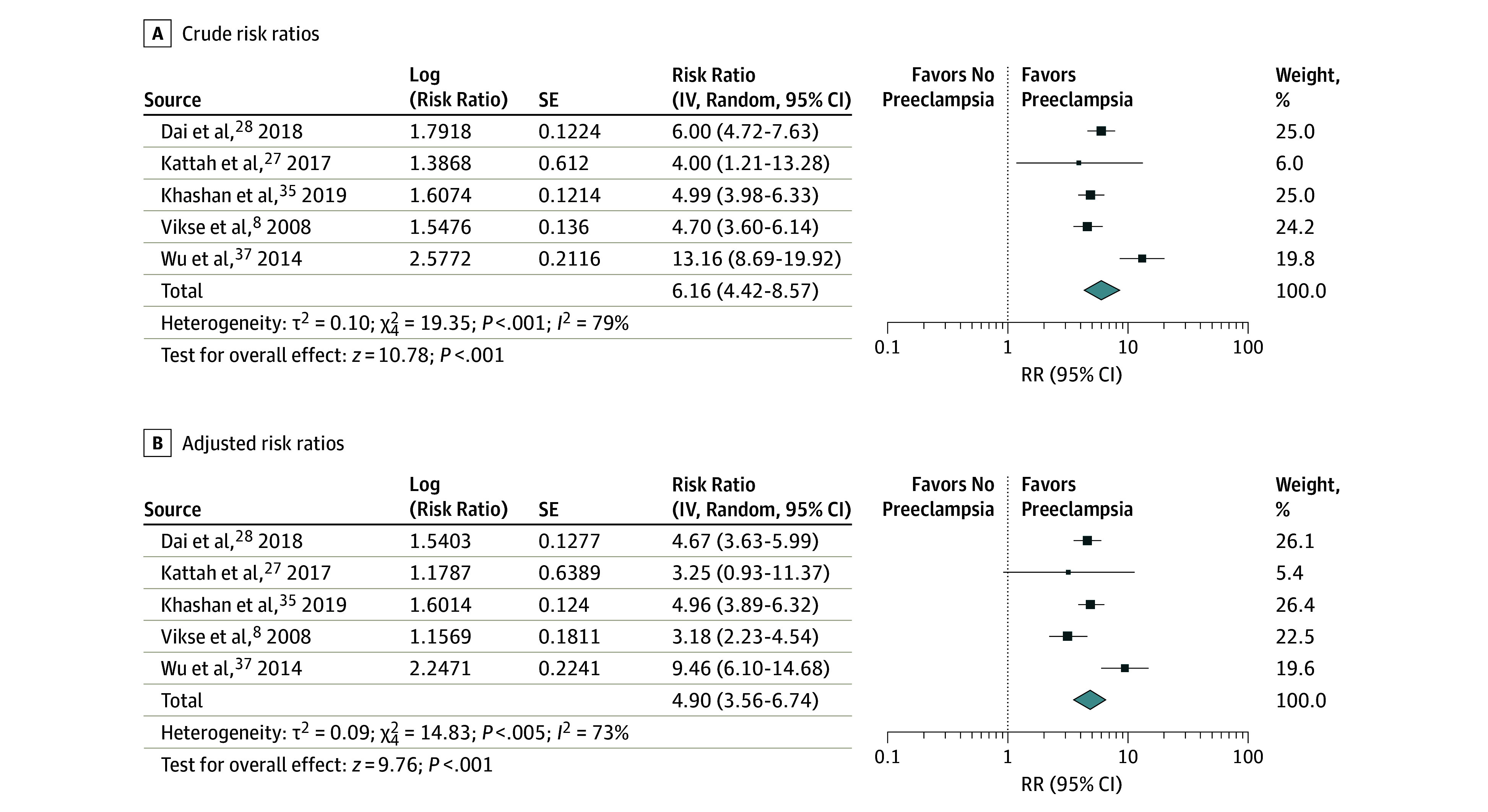
Forest Plot for Studies of the Association of Preeclampsia and End-Stage Kidney Disease Risk ratios (RRs) are calculated using random-effects meta-analysis. The area of the markers within the graph indicates the weight assigned to each study in the meta-analysis, while the horizontal line depicts the 95% CI. IV indicates inverse variance.

The pooled crude risk ratio for preeclampsia and ESKD was 6.16 (95% CI, 4.42-8.57) and the pooled adjusted risk ratio (aRR) was 4.90 (95% CI, 3.56-6.74) (Figure 2). High levels of heterogeneity were observed (*I*^2^ = 73%), owing to 1 study with outlying effect estimates.^37^ When this study was excluded, the pooled aRR for preeclampsia and ESKD was attenuated to 4.32 (95% CI, 3.50-5.32; *I*^2^ = 34%).

Three studies reported adjusted estimates for preeclampsia and CKD (pooled aRR, 2.11; 95% CI, 1.72-2.59) (eFigure 1 in the Supplement). Four studies reported associations between preeclampsia and kidney-related hospitalization. One study was excluded from meta-analysis owing to overlap between study populations.^32^ The pooled aRR for preeclampsia and kidney-related hospitalization was 2.65 (95% CI, 1.03-6.77). The population-attributable fraction for preeclampsia on ESKD was 11.4% and for CKD was 4.0%.

### Other HDP

The aRR for gestational hypertension and ESKD was 3.64 (95% CI, 2.34-5.66), but was based on only 2 effect estimates (eFigure 2 in the Supplement). Gestational hypertension was also associated with an increased risk of CKD (aRR, 1.49; 95% CI, 1.11-2.01). Two studies reported crude estimates for chronic hypertension and ESKD or for PSH and ESKD, and the pooled estimates suggested an increased risk of ESKD (chronic hypertension: RR, 16.87; 95% CI, 11.31-25.15; and PSH: RR, 48.97; 95% CI, 26.09-91.93), but were based on small numbers of patients.

### Preterm Delivery, Low Birth Weight, and GDM

Three studies reported adjusted effect estimates for preterm preeclampsia and ESKD (aRR, 5.66; 95% CI, 3.06-10.48) (eFigure 3 in the Supplement). Four studies reported adjusted effect estimates for preterm delivery and ESKD, independent of preeclampsia, but 1 was excluded owing to overlap between study populations^30^ (aRR, 2.09; 95% CI, 1.64-2.66, based on 3 effect estimates) (eFigure 4 in the Supplement).

When we further excluded 1 study restricted to women with preexisting diabetes,^29^ the association with ESKD was strengthened for both preterm preeclampsia (aRR, 7.51; 95% CI, 4.86-11.58) and preterm delivery alone (aRR, 2.24; 95% CI, 1.80-2.79).

Four studies reported associations between delivery of a low-birth-weight or growth-restricted infant (including infants who were small for gestational age or had intrauterine growth restriction) with ESKD or kidney-related hospitalization. Three studies reported an increased adjusted risk of ESKD or kidney-related hospitalization. However, these studies were not suitable for meta-analysis owing to excessive clinical and methodological diversity.

Three studies reported crude associations between GDM and CKD. When these effect estimates were pooled, no significant association was observed (RR, 0.99; 95% CI, 0.66-1.49) (eFigure 5 in the Supplement). Two of these studies provided adjusted estimates of CKD that could be combined in meta-analysis.^44,45^ No significant association was observed when the overall adjusted results were pooled. However, subgroup analysis showed an association with race/ethnicity: black women with GDM had a significantly higher risk of CKD (aRR, 1.78; 95% CI, 1.18-2.70), whereas white women did not (aRR, 0.81; 95% CI, 0.58-1.13) (eFigure 6 in the Supplement).

All meta-analyses were repeated using the Sidik-Jonkman method (eTable 4 in the Supplement). This method resulted in wider 95% CIs for most pooled estimates. The associations between preeclampsia and kidney-related hospitalization and preeclampsia and CKD were attenuated, but the latter remained statistically significant (aRR, 1.92; 95% CI, 1.17-3.14). The results were not otherwise meaningfully different.

## Discussion

The aim of this systematic review and meta-analysis was to synthesize the published literature on the association between adverse pregnancy outcomes and the risk of maternal CKD and ESKD. Overall, the literature in this area was sparse, but 4 principal findings emerged. First, our adjusted pooled results indicate that HDP are associated with long-term kidney disease, particularly ESKD. The type of HDP was important; both gestational hypertension and preeclampsia were associated with increased risk of maternal ESKD, but the association was stronger for preeclampsia, particularly preterm preeclampsia. Only 1 study provided adjusted effect estimates for women exposed to chronic hypertension or PSH,^37^ but the risk of ESKD was particularly high in these women. Mothers exposed to HDP are at elevated risk of cardiovascular and cerebrovascular disease,^5,49^ and our results suggest that their risk of long-term kidney disease is also elevated. The risk of maternal CKD after preeclampsia was lower than for ESKD, but 1 of the included studies on CKD had relatively small numbers of outcomes.^33^

Second, the adjusted pooled estimates suggest that preterm delivery may be independently associated with higher risk of maternal ESKD. However, this meta-analysis was based on only 3 studies. None of these studies considered whether preterm delivery was spontaneous or obstetrically indicated, although they had adjusted for preeclampsia.^8,28,29^ Pariente et al^43^ reported that the risk of kidney-related hospitalization was independently increased among Israeli women with preterm delivery, of whom most (70%) had spontaneous preterm delivery. When their analysis was restricted to those with indicated preterm delivery, no significant difference in kidney-related hospitalization was observed. Further research is warranted to elucidate whether the association between preterm delivery and CKD and ESKD differs by obstetric indication.

Third, existing literature suggests that delivery of infants with low birth weight or who are small for gestational age may be associated with increased risk of maternal kidney disease.^8,28,41^ However, there is a paucity of research examining this association, and only 1 of these studies controlled for maternal preeclampsia.^8^ It is possible that the apparent associations are confounded by unmeasured placental factors.

Fourth, it is not possible to conclude whether GDM is independently associated with an increased risk of maternal kidney disease based on the available literature. Although our meta-analysis suggests that GDM has no overall association with CKD risk, this finding was based on adjusted estimates from only 2 studies, and a subgroup analysis suggested that black women exposed to GDM have an increased risk of kidney disease. Black women have an increased risk of CKD compared with white women,^50,51,52^ and they are more likely to progress to ESKD.^53^ The reasons for this elevated risk remain poorly understood, but it is plausible that GDM itself may act as an independent factor associated with kidney disease. The 4 included studies on GDM differed in their treatment of subsequent type 2 diabetes; Bomback et al^45^ controlled for it by stratification and Dehmer et al^44^ treated it as a mediating factor, whereas neither of the 2 Israeli studies collected data on subsequent diabetes.^40,42^

### Interpretation and Mechanisms

The mechanisms underlying observed associations in this study are uncertain, and it is plausible that some adverse pregnancy outcomes are a manifestation of underlying predisposition to chronic disease. Irrespective of causality, our results suggest that adverse pregnancy outcomes may signal future risk of CKD and ESKD, particularly after HDP.

For preeclampsia and kidney disease, preexisting hypertension and obesity are risk factors that are common to both diseases, and these shared risk factors may partly explain this association. However, most studies in this systematic review reported strong associations even after controlling for, or excluding, women with prepregnancy hypertension^8,9,27,28,29,30,31,35,36,37,38,39^ or obesity.^28,32,33,35,39,42^

Preeclampsia has been proposed as a cause of lasting vascular endothelial dysfunction, possibly associated with elevated levels of soluble fms-like tyrosine kinase-1, an antiangiogenic protein secreted by the placenta that may remain elevated post partum.^1,54^ Higher levels of soluble fms-like tyrosine kinase-1 have been linked with progression of atherosclerosis^55^ and have also been observed among patients with CKD.^56^ Women exposed to preeclampsia may also experience arterial stiffness^57^ or lasting endothelial damage,^1^ which may be associated with their increased risk of future renal complications.^6^

The observed associations between preterm delivery or delivery of a low-birth-weight infant and maternal kidney disease may reflect subclinical disease or inflammatory processes. These associations may also be prone to residual confounding. Certain cardiovascular risk factors may be mutual to preterm delivery, growth restriction, and maternal CKD. For example, none of the studies that examined these associations had adjusted for maternal smoking, and only 1 effect estimate for preterm delivery and kidney disease was adjusted for maternal body mass index.^28^

The potential mechanisms by which GDM may increase the risk of kidney disease are more intuitive, because GDM can cause persistent endothelial dysfunction.^14,15^ However, smaller observational studies have examined the risk of early kidney damage in the years after pregnancy complicated by GDM, and results have been inconsistent to date.^16,58,59,60^

### Strengths and Limitations

To our knowledge, this is the most comprehensive systematic review and meta-analysis of adverse pregnancy outcomes and their associations with maternal kidney disease to date. An independent librarian verified the search strategy, and 3 relevant databases were included, supplemented by hand searching of reference lists. Most of the included studies had large sample sizes, were longitudinal, and had low risk of bias.

We observed high levels of heterogeneity in some of the meta-analyses, as measured using *I*^2^ and τ^2^ statistics. These measures should be interpreted with caution,^61^ and should not be considered substitutes for assessments of clinical or methodological diversity.^62^ We further excluded outlying effect estimates and conducted sensitivity analyses to reduce heterogeneity,^61^ but some clinical and methodological diversity persisted.

For meta-analyses involving CKD as the outcome variable, variation in ascertainment of the outcome may be associated with relatively high levels of heterogeneity. For example, some studies based their definitions of CKD on clinical criteria using estimated glomerular filtration rate values with or without albumin excretion rates.^32,34,39^ Others based their definitions on *International Classification of Diseases* registry codes, which were inconsistent between different studies.^9,33,38^ We were unable to conduct meaningful subgroup analyses owing to the small number of studies in each individual meta-analysis. Similarly, we were unable to assess for publication bias, owing to small numbers of included studies in each meta-analysis.

We excluded articles that reported microalbuminuria or other markers of kidney dysfunction as the only outcome, without clear information relating to CKD or ESKD. This decision was taken to maximize probability that those who were included in the review had a clinically significant diagnosis of CKD or ESKD, and not an incidental or screening-detected finding. Our review may not capture all articles with information relating to early, asymptomatic CKD. Stage 1 CKD may be diagnosed based on microalbuminuria alone,^63^ but it is of lesser clinical relevance, and its inclusion may have led to further heterogeneity in meta-analyses.

Some studies reporting secondary outcomes (hospitalization or mortality) did not clearly exclude women with preexisting kidney disease at baseline,^36,39,46,47^ which may have confounded associations. Furthermore, potential confounding by subclinical, undiagnosed nephropathy cannot be ruled out, even after excluding women with established kidney disease. Because the early stages of CKD are typically asymptomatic, it is possible that for some women, pregnancy unmasks an existing predisposition to kidney disease, rather than causing this predisposition de novo.

The adjusted effect estimates in our study were based on different definitions across individual studies. We considered maternal comorbidities to be the most important confounders, and we conducted sensitivity analyses based on studies that had adjusted for these comorbidities. A large nationwide cohort study of preeclampsia and ESKD reported no difference between crude and adjusted effect estimates after controlling for other maternal factors such as body mass index, smoking, and educational level.^35^ However, we cannot rule out the possibility of residual confounding by these (or other) maternal factors.

Finally, we used the generic inverse variance method (DerSimonian-Laird method) for meta-analysis.^62^ This method may produce biased effect estimates,^64^ and its CIs may have below-nominal coverage,^21^ particularly when the number of included studies is small. However, we repeated all meta-analyses using the Sidik-Jonkman method, which has lower error rates,^21^ and the results were not meaningfully different.

### Implications and Future Research

The findings in this systematic review may be of substantial public health relevance. The prevalence of adverse pregnancy outcomes, including HDP, has been increasing,^65,66,67^ and the worldwide prevalence of stage 3 or greater CKD is now estimated at 12% among women.^68^ Our study estimates that 11% of all cases of maternal ESKD and 4% of cases of CKD may be associated with preeclampsia alone.

Clinical guidelines have highlighted the need for women’s obstetric history to be routinely used in their risk stratification and prevention of cardiometabolic disease.^5,69^ The findings of this study may be used to inform clinical prediction tools for physicians to stratify which women need closer follow-up for CKD. The absolute risk of clinically significant kidney disease may remain low for exposed women, but a systematic approach may be warranted to identify and advise those who are at increased relative risk, particularly after HDP. However, further robust research is needed to overcome limitations of the existing literature, and to identify effective risk reduction interventions.

## Conclusions

Women who experience adverse pregnancy outcomes may be at increased risk of future CKD and ESKD. The associations appear to be particularly marked for women with HDP. It is unclear whether adverse pregnancy outcomes unmask an existing predisposition toward kidney disease or induce endothelial or organ damage that alters a woman’s trajectory toward development of kidney disease. There is a need to optimize long-term follow-up of these women, and to implement preventive interventions that reduce their risk of developing clinically significant kidney disease.

## Notes

Supplement. **eTable 1.** Characteristics of Studies Which Investigate Preterm Delivery and Subsequent Maternal Renal Disease **eTable 2.** Characteristics of Studies Which Investigate Delivery of a Low Birth Weight or Small for Gestational Age Infant and Subsequent Maternal Renal Disease **eTable 3.** Characteristics of Studies Which Investigate Gestational Diabetes and Subsequent Maternal Renal Disease **eTable 4.** Summary Results of Post Hoc Meta-analyses Based on Sidik-Jonkman Method **eFigure 1.** Forest Plot for Studies of the Association of Preeclampsia and Chronic Kidney Disease **eFigure 2.** Forest Plot for Studies of the Association of Gestational Hypertension And End-stage Kidney Disease **eFigure 3.** Forest Plot for Studies of the Association of Gestational Hypertension and Chronic Kidney Disease **eFigure 4.** Forest Plot for Studies of the Association of Preterm Preeclampsia and End-stage Kidney Disease **eFigure 5.** Forest Plot for Studies of the Association of Preterm Delivery and End-stage Kidney Disease **eFigure 6.** Forest Plot for Studies of the Adjusted Association of Gestational Diabetes and Chronic Kidney Disease
