# Supplementary material for: Adverse Pregnancy Outcomes and Long-term Maternal Kidney Disease: A Systematic Review and Meta-analysis
Source: JAMA Netw Open. 2020 Feb 12;3(2):e1920964. doi: 10.1001/jamanetworkopen.2019.20964 (PMC12527481; doi:10.1001/jamanetworkopen.2019.20964)
Supplement: Supplement. — eTable 1. Characteristics of Studies Which Investigate Preterm Delivery and Subsequent Maternal Renal Disease eTable 2. Characteristics of Studies Which Investigate Delivery of a Low Birth Weight or Small for Gestational Age Infant and Subsequent Maternal Renal Disease eTable 3. Characteristics of Studies Which Investigate Gestational Diabetes and Subsequent Maternal Renal Disease eTable 4. Summary Results of Post Hoc Meta-analyses Based on Sidik-Jonkman Method eFigure 1. Forest Plot for Studies of the Association of Preeclampsia and Chronic Kidney Disease eFigure 2. Forest Plot for Studies of the Association of Gestational Hypertension And End-stage Kidney Disease eFigure 3. Forest Plot for Studies of the Association of Gestational Hypertension and Chronic Kidney Disease eFigure 4. Forest Plot for Studies of the Association of Preterm Preeclampsia and End-stage Kidney Disease eFigure 5. Forest Plot for Studies of the Association of Preterm Delivery and End-stage Kidney Disease eFigure 6. Forest Plot for Studies of the Adjusted Association of Gestational Diabetes and Chronic Kidney Disease [file jamanetwopen-e1920964-s001.pdf]

Supplementary Online Content

Barrett PM, McCarthy FP, Kublickiene K, et al. Adverse pregnancy outcomes and long-term maternal kidney disease: a systematic review and meta-analysis. *JAMA Netw Open.* 2020;3(2):e1920964. doi:10.1001/jamanetworkopen.2019.20964

eTable 1. Characteristics of Studies Which Investigate Preterm Delivery and Subsequent Maternal Renal Disease
eTable 2. Characteristics of Studies Which Investigate Delivery of a Low Birth Weight or Small for Gestational Age Infant and Subsequent Maternal Renal Disease
eTable 3. Characteristics of Studies Which Investigate Gestational Diabetes and Subsequent Maternal Renal Disease
eTable 4. Summary Results of Post Hoc Meta-analyses Based on Sidik-Jonkman Method
eFigure 1. Forest Plot for Studies of the Association of Preeclampsia and Chronic Kidney Disease
eFigure 2. Forest Plot for Studies of the Association of Gestational Hypertension and End-stage Kidney Disease
eFigure 3. Forest Plot for Studies of the Association of Gestational Hypertension and Chronic Kidney Disease
eFigure 4. Forest Plot for Studies of the Association of Preterm Preeclampsia and End-stage Kidney Disease
eFigure 5. Forest Plot for Studies of the Association of Preterm Delivery and End-stage Kidney Disease
eFigure 6. Forest Plot for Studies of the Adjusted Association of Gestational Diabetes and Chronic Kidney Disease

This supplementary material has been provided by the authors to give readers additional information about their work.

© 2020 Barrett PM et al. *JAMA Network Open.*

eTable 1. Characteristics of Studies Which Investigate Preterm Delivery and Subsequent Maternal Renal Disease

Author, year published	Country, *Follow-up*	Study design*, Data source	Sample size	Exposure(s)	Outcome(s), *Measure of effect*	Exclusions	Confounders adjusted	Crude Risk Ratio (95% CI)	Adjusted Risk Ratio (95% CI)
**Dai, 2018 (28)**	Canada, *median 15 years*	RC, Hospital records	1598043	Preterm delivery	ESKD hospitalization, *HR*	Maternal age <15 or >44 years, multiple gestation, previous kidney disease, DM, GDM, SLE, HUS, thrombotic microangiopathy, hypertension secondary to kidney disease.	Maternal age, region, time period, obesity, preterm delivery, intrauterine death, fetal distress, placental disorders/abruption, oligohydramnios, prolonged pregnancy, postpartum haemorrhage, DVT, cardiac disease, blood transfusion, caesarean delivery	*3.50 (2.57-4.77) based on restricted analysis of women with only one pregnancy*	Overall (based on index pregnancy) 2.36 (1.81–3.08); *restricted to women who only had one pregnancy, 2.42 (1.75-3.35)*
**Sandvik, 2010 (29)**	Norway, *up to 37 years*	RC, Norwegian Renal Registry	1481	Preterm delivery	ESKD, *RR*	Multiple deliveries, previous kidney disease, hypertension. All included women had pre-existing diabetes.	Year of birth, age, marital status, stillbirth, congenital malformations of offspring, Caesarean section in first pregnancy	1.9 (1.1-3.4)	1.4 (0.71-2.6)
**Vikse, 2008 (8)**	Norway, *mean 27 years*	RC, Norwegian Renal Registry	570433	PE	ESKD, *RR*	Multiple pregnancies, previous kidney disease, hypertension, rheumatic disease or DM	Year of delivery, maternal age, marital status, stillbirth, congenital malformation of infant	3.8 (2.9-4.9)	2.0 (1.4-3.0)
**Vikse, 2010 (30)**	Norway, *up to 16 years*	RC, Norwegian Renal Registry	582	Preterm delivery	ESKD, *RR*	None specified, but study restricted to women who underwent renal biopsy for suspected renal damage	Maternal age, eGFR, proteinuria, diastolic blood pressure, duration of renal disease, interstitial fibrosis and inflammation	2.1 (1.2–3.9)	2.4 (1.2–4.6)
**Pariente, 2017 (43)**	Israel, *mean 11 years*	RC, Clinical records at single institution	99338	Preterm delivery	Renal hospitalization, *HR*	Previous renal disease, congenital renal malformations	PE, DM, indicated preterm delivery (due to severe PE, severe IUGR, cord prolapse or placental abruption)	Overall preterm delivery, 2.9 (2.0-4.2); spontaneous preterm, 2.6 (1.7-3.9); induced preterm, 4.2 (2.0-9.1); indicated preterm 3.4 (1.7-6.5)	Overall preterm delivery, 2.7 (1.8-3.9); indicated preterm, 1.2 (0.7-1.9)

*Study design: RC, retrospective cohort

CI, confidence interval; DM, diabetes mellitus; DVT, deep vein thrombosis; eGFR, estimated glomerular filtration rate; ESKD, end-stage kidney disease; GDM, gestational diabetes mellitus; HUS, hemolytic uremic syndrome; IUGR, intra-uterine growth restriction; RR, risk ratio; PE, preeclampsia; SLE, systemic lupus erythematosus

© 2020 Barrett PM et al. *JAMA Network Open.*

eTable 2. Characteristics of Studies Which Investigate Delivery of a Low Birth Weight or Small for Gestational Age Infant and Subsequent Maternal Renal Disease

Author, year published	Country, Follow-up	Study design*, Data source	Sample size	Exposure(s)	Outcome(s), *Measure of effect*	Exclusions	Confounders adjusted	Crude Risk Ratio (95% CI)	Adjusted Risk Ratio (95% CI)
**Vikse, 2008 (8)**	Norway, *mean 27 years*	RC, Norwegian Renal Registry	570433	Low birth weight	ESKD, *RR*	Multiple pregnancies, previous kidney disease, hypertension, rheumatic disease or DM	Year of delivery, maternal age, marital status, stillbirth, congenital malformation of infant	In women with no PE, 4.0 (3.0–5.2); women with PE, 12.0 (8.2–17.6)	In women with no PE, 2.7 (1.8–3.8); women with PE, 6.8 (3.9–12.0)
**Vikse, 2010 (30)**	Norway, *up to 16 years*	RC, Norwegian Renal Registry	582	Low birth weight	ESKD, *RR*	None specified, but study restricted to women who underwent renal biopsy for suspected renal damage	Maternal age, eGFR, proteinuria, diastolic blood pressure, duration of renal disease, interstitial fibrosis and inflammation	1.7 (0.92–3.2)	1.7 (0.83–3.6)
**Dai, 2018 (28)**	Canada, *median 15 years*	RC, Hospital records	1598043	IUGR	ESKD hospitalization, *HR*	Maternal age <15 or >44 years, multiple gestation, previous kidney disease, DM, GDM, SLE, HUS, thrombotic microangiopathy, hypertension secondary to kidney disease.	Maternal age, region, time period, obesity, preterm delivery, intrauterine fetal death, fetal distress, placenta disorders/abruption, oligohydramnios, prolonged pregnancy, postpartum haemorrhage, deep vein thrombosis and cardiac disease, blood transfusion, caesarean delivery	*3.47 (2.32–5.18) based on restricted analysis of women with only one pregnancy*	Overall (based on index pregnancy) 1.91 (1.34–2.71); restricted to women who only had one pregnancy, 1.98 (1.30-3.02)
**Almasi, 2016 (41)**	Israel, *mean 11 years*	RC, Clinical records at a single institution	99342	SGA	Renal hospitalization, *HR*	Women with multiple pregnancies, pre-existing kidney disease, DM, hypertension, not engaged in antenatal care	GDM, number of pregnancies	1.6 (1.02-2.60)	1.79 (1.10-2.80)

*Study design: RC, retrospective cohort

CI, confidence interval; DM, diabetes mellitus; DVT, deep vein thrombosis; eGFR, estimated glomerular filtration rate; ESKD, end-stage kidney disease; GDM, gestational diabetes mellitus; HR, hazard ratio; HUS, hemolytic uremic syndrome; IUGR, intra-uterine growth restriction; OR, odds ratio; PE, preeclampsia; RR, risk ratio; SGA, small for gestational age; SLE, systemic lupus erythematosus

© 2020 Barrett PM et al. *JAMA Network Open.*

eTable 3. Characteristics of Studies Which Investigate Gestational Diabetes and Subsequent Maternal Renal Disease

Author, year published	Country, *Follow-up*	Study design*, Data source	Sample size	Exposure(s)	Outcome(s), *Measure of effect*	Exclusions	Confounders adjusted	Crude Risk Ratio (95% CI)	Adjusted Risk Ratio (95% CI)
**Kessous, 2015 (42)**	Israel, *mean 11 years*	RC, Clinical records at single institution	96370	GDM	Renal hospitalization, *HR*	Previous cardiovascular or renal disease, congenital cardiac or renal malformations, multiple pregnancy	Maternal age, parity, obesity, smoking. (Unclear whether adjusted for interim DM)	Not reported	1.9 (1.1-3.2)
**Dehmer, 2018 (44)**	USA, *mean 21 years*	PC, Participants in CARDIA cohort study	820	GDM	CKD, *HR*	Previous CKD, DM, women missing measures of baseline CKD/albuminuria/ eGFR, women missing data on covariates	Maternal age, systolic blood pressure, dyslipidemia, BMI, smoking, education, eGFR, fasting glucose, physical activity level, race, family history DM. (DM treated as mediating factor)	1.46 (0.87-2.45)	For all: 1.33 (0.78-2.26) Black: 1.96 (1.04-3.67) White: 0.65 (0.23-1.83)
**Beharier, 2015 (40)**	Israel, *mean 11 years*	RC, Clinical records at a single institution	97,968	GDM	Renal-related hospitalization, CKD, ESKD, *OR*	Multiple pregnancies, previous kidney disease, pregnancies with missing data on key variables relating to prenatal care	Maternal age, parity. (No data on interim DM)	For total renal morbidity: 2.34 (1.4-3.7). For CKD, 0.73 (0.3-2.2). For ESKD, 1.14 (0.2-5.9)	For total renal morbidity 1.70 (1.05-2.60)
**Bomback, 2010 (45)**	USA	PC, Participant records from community screening programme	37716	GDM	CKD (Stages 1-2, & stages 3-5), *OR*	Women with previous ESKD	Maternal age, race, BMI, current smoking, alcohol use, hypertension, dyslipidemia, family history of kidney disease. (Stratified by DM status)	Among those without subsequent DM: CKD stages 1-2, 1.54 (1.16–2.05); stages 3-5, 0.84 (0.65–1.09).	Among those without subsequent DM: CKD stages 1–2, 1.54 (1.16–2.05); White, 1.12 (0.68-1.84); African-American, 2.32 (1.50-3.60); CKD stages 3-5, 0.94 (0.71-1.25); White 0.83 (0.58-1.19); African-American 1.66 (0.96-2.88)

*Study design: PC, prospective cohort; RC, retrospective cohort

BMI, body mass index; CI, confidence interval; CKD, chronic kidney disease; DM, diabetes mellitus; eGFR, estimated glomerular filtration rate; ESKD, end-stage kidney disease; GDM, gestational diabetes mellitus; HR, hazard ratio; OR, odds ratio; PE, preeclampsia

© 2020 Barrett PM et al. *JAMA Network Open.*

eTable 4. Summary Results of Post Hoc Meta-analyses Based on Sidik-Jonkman Method

Exposure	Outcome	No. of studies	References	Participants	Total no. of outcomes	Pooled RR (95% CI)	I^2, %	Tau^2
**Preeclampsia**	**ESKD**							
	Crude	5	(8, 27, 28, 35, 37)	4,479,523	1,737	6.17 (4.18-9.10)	85%	0.15
	Adjusted (any)	5	(8, 27, 28, 35, 37)	4,479,523	1,737	4.90 (3.44-6.98)	78%	0.11
	Adjusted for comorbidities	5	(8, 27, 28, 35, 37)	4,479,523	1,737	4.90 (3.44-6.98)	78%	0.11
	**CKD**							
	Crude	3	(9, 32, 33)	1,097,495	4,699	1.98 (0.88-4.45)	95%	0.41
	Adjusted (any)	3	(9, 32, 33)	1,097,495	4,699	1.92 (1.17-3.14)	84%	0.13
	Adjusted for comorbidities	1	(9)	1,072,330	3,901	2.27 (2.02-2.55)	-	-
	**Kidney-related hospitalization**							
	Crude	2	(36, 42)	131,224	468	1.80 (0.64-5.02)	94%	0.52
	Adjusted (any)	3	(36, 42, 47)	162,880	1,051	2.66 (0.96-7.40)	93%	0.74
	Adjusted for comorbidities	0	-	-	-	-	-	-
**Gestational hypertension**	**ESKD**							
	Crude	2	(28, 37)	2,542,517	806	4.43 (1.65-11.84)	71%	0.37
	Adjusted (any)	2	(28, 37)	2,542,517	806	3.64 (2.34-5.66)	7%	0.01
	Adjusted for comorbidities	2	(28, 37)	2,542,517	806	3.64 (2.34-5.66)	7%	0.01
	**CKD**							
	Crude	2	(32, 33)	25,165	798	1.57 (1.07-2.29)	59%	0.05
	Adjusted (any)	2	(32, 33)	25,165	798	1.50 (1.10-2.04)	43%	0.03
	Adjusted for comorbidities	0	-	-	-	-	-	-
	**Kidney-related hospitalization**							
	Crude	2	(32, 36)	49,705	1,010	1.04 (0.92-1.17)	0%	0.00
	Adjusted (any)	2	(36, 47)	66,510	939	1.88 (0.44-7.94)	94%	1.02
	Adjusted for comorbidities	0	-	-	-	-	-	-
**Chronic hypertension**	**ESKD**							
	Crude	2	(28, 37)	2,542,517	806	16.87 (11.31-25.15)	0%	0.00
	Adjusted (any)	1	(37)	944,474	258	15.99 (5.89-43.41)	-	-
	Adjusted for comorbidities	1	(37)	944,474	258	15.99 (5.89-43.41)	-	-
	**CKD**							
	Crude	1	(33)	10,314	144	1.62 (0.88-3.00)-	-	-
	Adjusted (any)	1	(33)	10,314	144	1.23 (0.67-2.26)	-	-
	Adjusted for comorbidities	0	-	-	-	-	-	-
**Superimposed preeclampsia**	**ESKD**							
	Crude	2	(28, 37)	2,542,517	806	48.97 (26.09-91.94)	41%	0.09

© 2020 Barrett PM et al. *JAMA Network Open.*

	Adjusted (any)	1	(37)	944,474	258	44.72 (22.59-88.52)	-	-
	Adjusted for comorbidities	1	(37)	944,474	258	44.72 (22.59-88.52)	-	-
	**CKD**							
	Crude	1	(33)	10,314	144	1.56 (0.38-6.42)-	-	-
	Adjusted (any)	1	(33)	10,314	144	1.24 (0.28-5.49)	-	-
	Adjusted for comorbidities	0	-	-	-	-	-	-
**Preterm delivery (no preeclampsia)**	**ESKD**							
	Crude	3	(8, 28, 29)	2,169,957	1,073	3.11 (2.05-4.72)	76%	0.10
	Adjusted (any)	3	(8, 28, 29)	2,169,957	1,073	2.06 (1.56-2.71)	32%	0.02
	Adjusted for comorbidities	3	(8, 28, 29)	2,169,957	1,073	2.06 (1.56-2.71)	32%	0.02
	**CKD**							
	Crude	0	-	-	-	-	-	-
	Adjusted (any)	0	-	-	-	-	-	-
	**Kidney-related hospitalization**							
	Crude	1	(43)	99,338	132	2.90 (2.00-4.20)	-	-
	Adjusted (any)	1	(43)	99,338	132	2.70 (1.80-3.90)	-	-
	Adjusted for comorbidities	0	-	-	-	-	-	-
**Preterm preeclampsia**	**ESKD**							
	Crude	3	(8, 29, 35)	1,938,355	935	7.56 (2.69-21.21)	90%	0.74
	Adjusted (any)	3	(8, 29, 35)	1,938,355	935	5.64 (2.99-10.67)	62%	0.19
	Adjusted for comorbidities	3	(8, 29, 35)	1,938,355	935	5.64 (2.99-10.67)	62%	0.19
	**CKD**							
	Crude	0	-	-	-	-	-	-
	Adjusted (any)	1	(9)	1,072,330	3,901	3.93 (2.90-5.33)	-	-
	Adjusted for comorbidities	1	(9)	1,072,330	3,901	3.93 (2.90-5.33)	-	-
**Gestational diabetes**	**ESKD**							
	Crude	0	-	-	-	-	-	-
	Adjusted (any)	0	-	-	-	-	-	-
	**CKD**							
	Crude	3	(40, 44, 45)	136,504	6,345	0.94 (0.73-1.21)	7%	0.01
	Adjusted (any)	2	(44, 45)	38,536	6,231	1.04 (0.76-1.41)	21%	0.01
	Adjusted for comorbidities	2	(44, 45)	38,536	6,231	1.04 (0.76-1.41)	21%	0.01
	**Kidney-related hospitalization**							
	Crude	0	-	-	-	-	-	-
	Adjusted (any)	1	(42)	96,370	112	1.90 (1.10-3.20)	-	-
	Adjusted for comorbidities	0	-	-	-	-	-	-

© 2020 Barrett PM et al. *JAMA Network Open.*

Meta-analysis was based on the Sidik-Jonkman method in Stata version 15.0. The log risk ratio (RR) and standard error for each study was entered in meta-analysis using the admetan command.

© 2020 Barrett PM et al. *JAMA Network Open.*

Crude

Study or Subgroup	log[Risk Ratio]	SE	Weight	Risk Ratio IV, Random, 95% CI
Ayansina et al. 2016	0.7031	0.1418	42.4%	2.02 [1.53, 2.67]
Kristensen et al. 2019	1.0986	0.0595	49.8%	3.00 [2.67, 3.37]
Mannisto et al. 2013	-0.3025	0.7195	7.8%	0.74 [0.18, 3.03]
**Total (95% CI)**			**100.0%**	**2.27 [1.48, 3.49]**

Heterogeneity: Tau² = 0.09; Chi² = 10.07, df = 2 (P = 0.006); I² = 80%
Test for overall effect: Z = 3.77 (P = 0.0002)

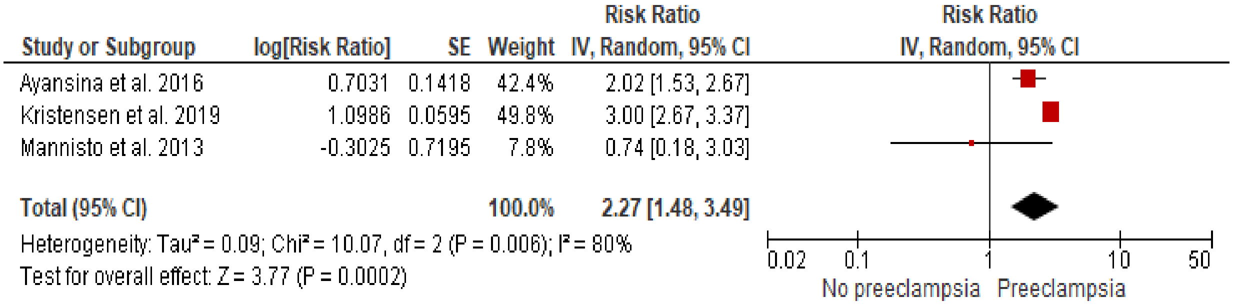

Adjusted

Study or Subgroup	log[Risk Ratio]	SE	Weight	Risk Ratio IV, Random, 95% CI
Ayansina et al. 2016	0.6539	0.1455	32.0%	1.92 [1.45, 2.56]
Kristensen et al. 2019	0.8198	0.0595	66.1%	2.27 [2.02, 2.55]
Mannisto et al. 2013	-0.2837	0.7654	1.8%	0.75 [0.17, 3.38]
**Total (95% CI)**			**100.0%**	**2.11 [1.72, 2.59]**

Heterogeneity: Tau² = 0.01; Chi² = 3.09, df = 2 (P = 0.21); I² = 35%
Test for overall effect: Z = 7.13 (P < 0.00001)

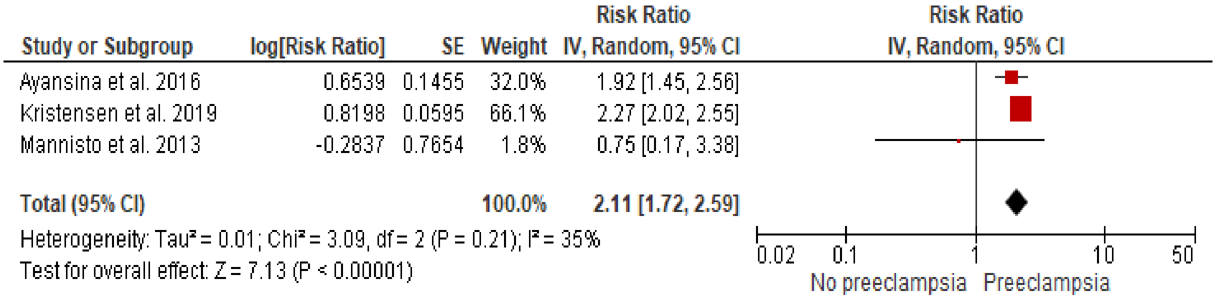

eFigure 1. Forest Plot for Studies of the Association of Preeclampsia and Chronic Kidney Disease

Risk ratios (RRs) are calculated using random-effects meta-analysis.

© 2020 Barrett PM et al. *JAMA Network Open.*

Crude

Study or Subgroup	log[Risk Ratio]	SE	Weight	Risk Ratio IV, Random, 95% CI
Dai et al. 2018	1.0986	0.233	60.8%	3.00 [1.90, 4.74]
Wu et al. 2014	2.0605	0.5046	39.2%	7.85 [2.92, 21.10]
**Total (95% CI)**			**100.0%**	**4.37 [1.74, 10.98]**

Heterogeneity: $Tau^2 = 0.31$; $Chi^2 = 3.00$, $df = 1$ ($P = 0.08$); $I^2 = 67\%$

Test for overall effect: $Z = 3.14$ ($P = 0.002$)

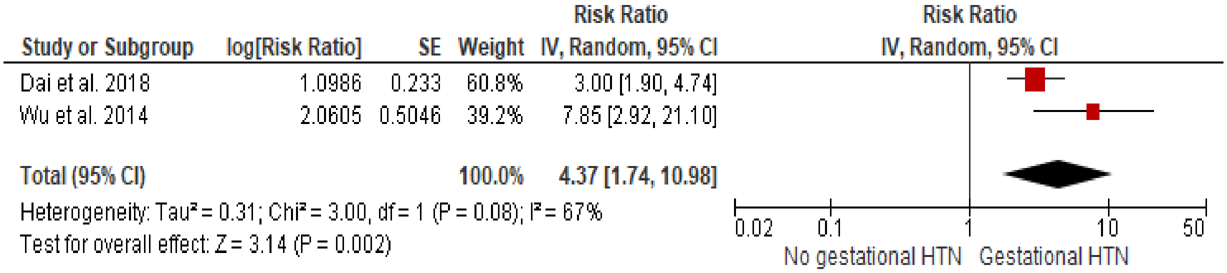

Adjusted

Study or Subgroup	log[Risk Ratio]	SE	Weight	Risk Ratio IV, Random, 95% CI
Dai et al. 2018	1.1848	0.2259	81.2%	3.27 [2.10, 5.09]
Wu et al. 2014	1.7613	0.5086	18.8%	5.82 [2.15, 15.77]
**Total (95% CI)**			**100.0%**	**3.64 [2.34, 5.66]**

Heterogeneity: $Tau^2 = 0.01$; $Chi^2 = 1.07$, $df = 1$ ($P = 0.30$); $I^2 = 7\%$

Test for overall effect: $Z = 5.74$ ($P < 0.00001$)

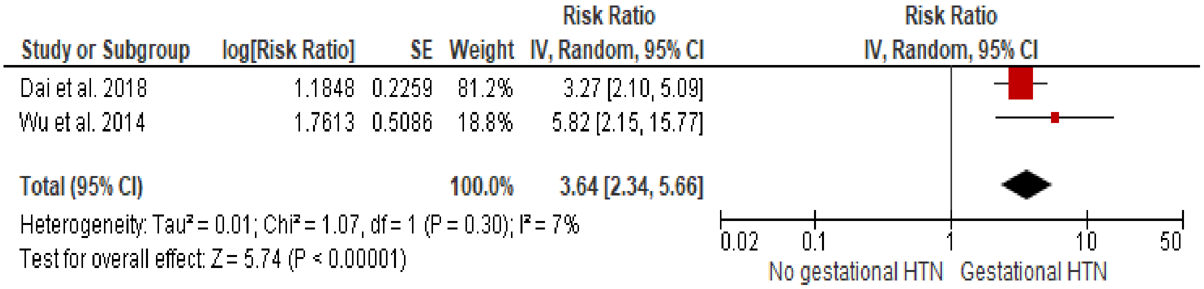

eFigure 2. Forest Plot for Studies of the Association of Gestational Hypertension and End-stage Kidney Disease

Risk ratios (RRs) are calculated using random-effects meta-analysis.

© 2020 Barrett PM et al. *JAMA Network Open.*

Crude

Study or Subgroup	log[Risk Ratio]	SE	Weight	Risk Ratio IV, Random, 95% CI
Ayansina et al. 2016	0.3148	0.0906	67.2%	1.37 [1.15, 1.64]
Mannisto et al. 2013	0.7031	0.2449	32.8%	2.02 [1.25, 3.26]
**Total (95% CI)**			**100.0%**	**1.56 [1.09, 2.22]**

Heterogeneity: Tau² = 0.04; Chi² = 2.21, df = 1 (P = 0.14); I² = 55%
Test for overall effect: Z = 2.43 (P = 0.02)

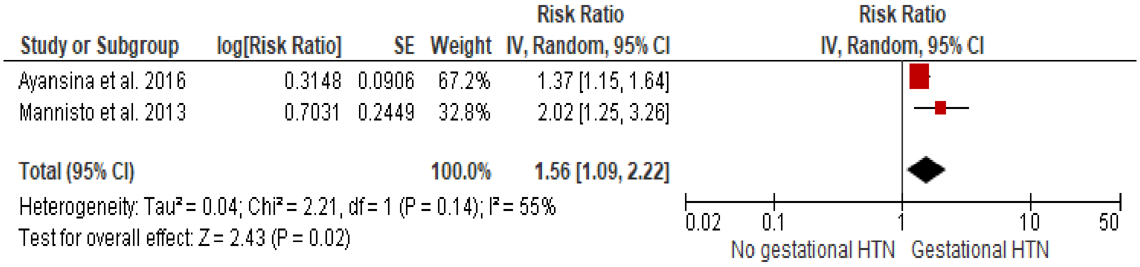

Adjusted

Study or Subgroup	log[Risk Ratio]	SE	Weight	Risk Ratio IV, Random, 95% CI
Ayansina et al. 2016	0.3075	0.0914	72.6%	1.36 [1.14, 1.63]
Mannisto et al. 2013	0.6471	0.2457	27.4%	1.91 [1.18, 3.09]
**Total (95% CI)**			**100.0%**	**1.49 [1.11, 2.01]**

Heterogeneity: Tau² = 0.02; Chi² = 1.68, df = 1 (P = 0.20); I² = 40%
Test for overall effect: Z = 2.64 (P = 0.008)

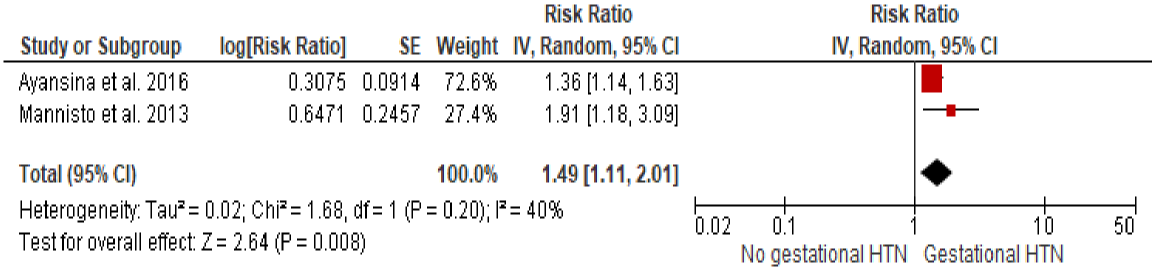

eFigure 3. Forest Plot for Studies of the Association of Gestational Hypertension and Chronic Kidney Disease

Risk ratios (RRs) are calculated using random-effects meta-analysis.

© 2020 Barrett PM et al. *JAMA Network Open.*

Crude

Study or Subgroup	log[Risk Ratio]	SE	Weight	Risk Ratio IV, Random, 95% CI
Khashan et al. 2019	2.2181	0.2941	33.6%	9.19 [5.16, 16.35]
Sandvik et al. 2010	1.0296	0.3915	30.2%	2.80 [1.30, 6.03]
Vikse et al. 2008	2.7081	0.2069	36.2%	15.00 [10.00, 22.50]
**Total (95% CI)**			**100.0%**	**7.66 [3.16, 18.55]**

Heterogeneity: Tau^2 = 0.52; Chi^2 = 14.50, df = 2 (P = 0.0007); I^2 = 86%
Test for overall effect: Z = 4.51 (P < 0.00001)

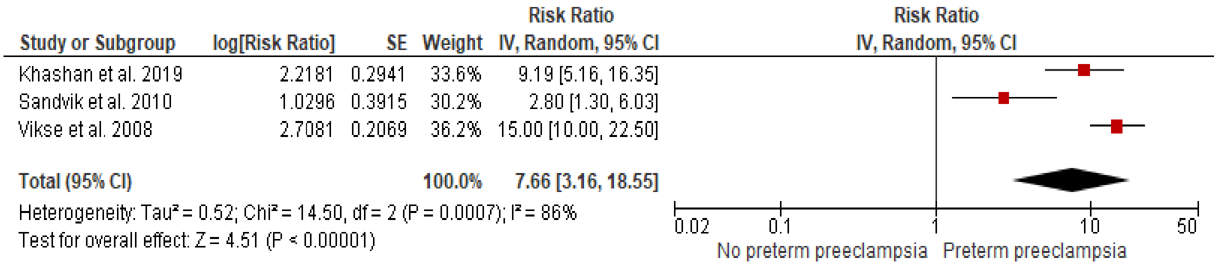

Adjusted

Study or Subgroup	log[Risk Ratio]	SE	Weight	Risk Ratio IV, Random, 95% CI
Khashan et al. 2019	2.1701	0.2949	37.8%	8.76 [4.91, 15.61]
Sandvik et al. 2010	1.0647	0.4055	29.2%	2.90 [1.31, 6.42]
Vikse et al. 2008	1.8245	0.3537	33.0%	6.20 [3.10, 12.40]
**Total (95% CI)**			**100.0%**	**5.66 [3.06, 10.48]**

Heterogeneity: Tau^2 = 0.17; Chi^2 = 4.87, df = 2 (P = 0.09); I^2 = 59%
Test for overall effect: Z = 5.52 (P < 0.00001)

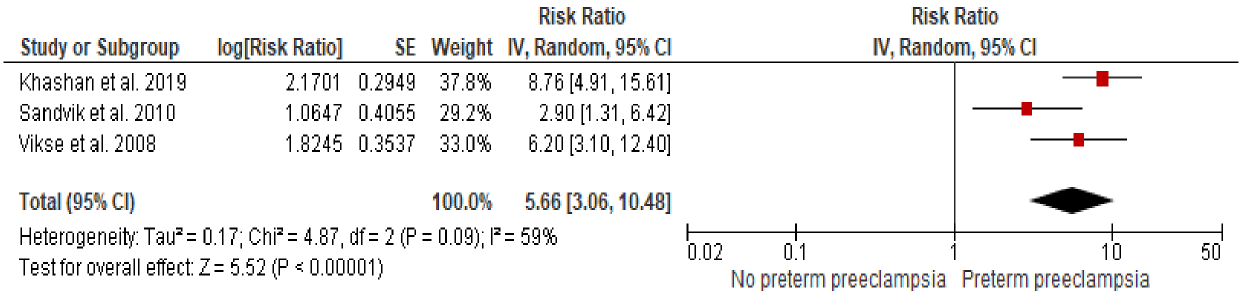

eFigure 4. Forest Plot for Studies of the Association of Preterm Preeclampsia and End-stage Kidney Disease

Risk ratios (RRs) are calculated using random-effects meta-analysis.

© 2020 Barrett PM et al. *JAMA Network Open.*

Crude

Study or Subgroup	log[Risk Ratio]	SE	Weight	Risk Ratio IV, Random, 95% CI
Dai et al. 2018	1.2528	0.1576	37.8%	3.50 [2.57, 4.77]
Sandvik et al. 2010	0.6419	0.293	19.8%	1.90 [1.07, 3.37]
Vikse et al. 2008	1.335	0.1327	42.3%	3.80 [2.93, 4.93]
**Total (95% CI)**			**100.0%**	**3.21 [2.35, 4.39]**

Heterogeneity: Tau² = 0.04; Chi² = 4.68, df = 2 (P = 0.10); I² = 57%
Test for overall effect: Z = 7.31 (P < 0.00001)

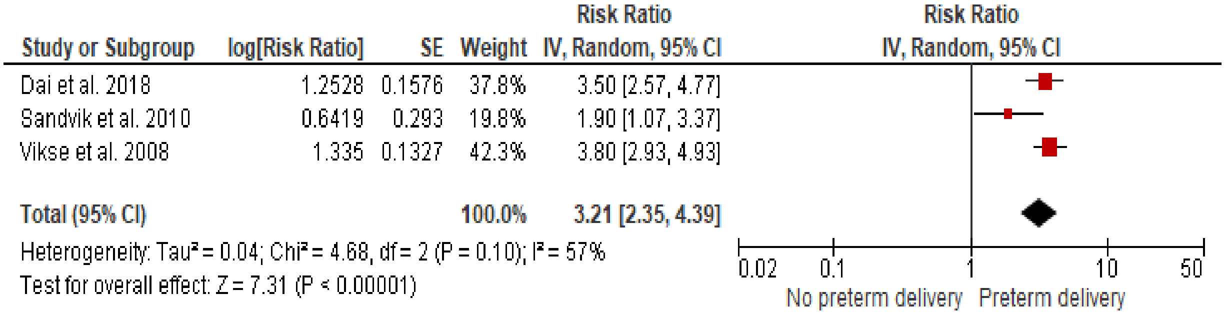

Adjusted

Study or Subgroup	log[Risk Ratio]	SE	Weight	Risk Ratio IV, Random, 95% CI
Dai et al. 2018	0.8587	0.1354	55.7%	2.36 [1.81, 3.08]
Sandvik et al. 2010	0.3148	0.3325	12.8%	1.37 [0.71, 2.63]
Vikse et al. 2008	0.6931	0.1986	31.5%	2.00 [1.36, 2.95]
**Total (95% CI)**			**100.0%**	**2.09 [1.64, 2.66]**

Heterogeneity: Tau² = 0.01; Chi² = 2.44, df = 2 (P = 0.30); I² = 18%
Test for overall effect: Z = 5.95 (P < 0.00001)

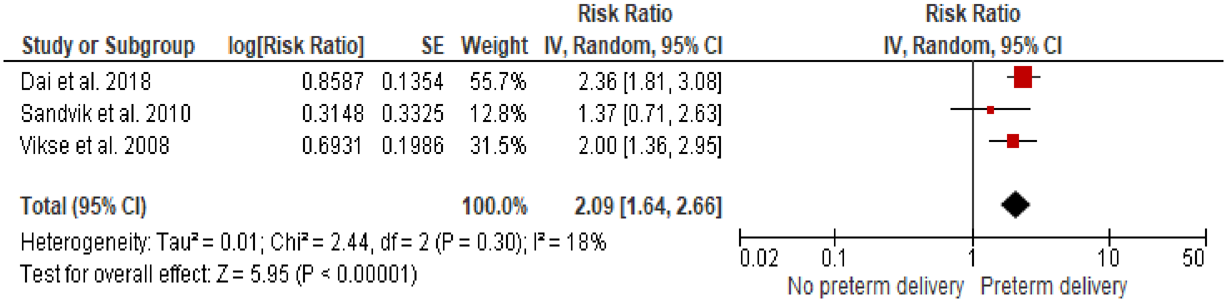

eFigure 5. Forest Plot for Studies of the Association of Preterm Delivery and End-stage Kidney Disease

Risk ratios (RRs) are calculated using random-effects meta-analysis.

© 2020 Barrett PM et al. *JAMA Network Open.*

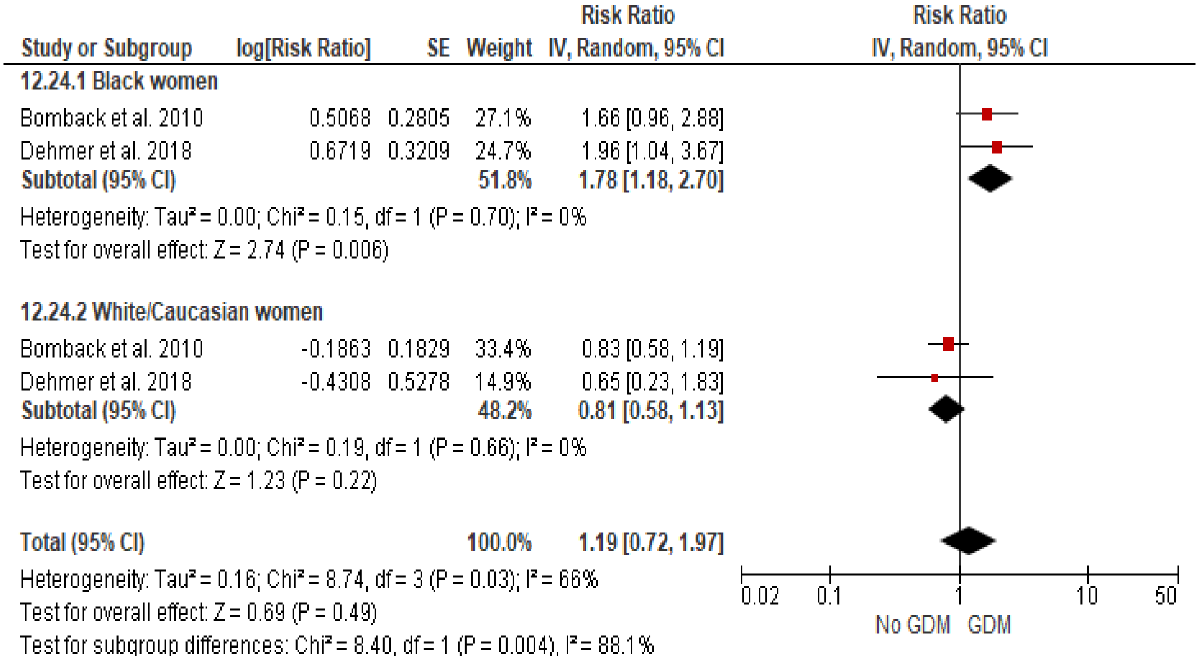

eFigure 6. Forest Plot for Studies of the Adjusted Association of Gestational Diabetes and Chronic Kidney Disease

Adjusted risk ratios (RRs) are calculated using random-effects meta-analysis.

© 2020 Barrett PM et al. *JAMA Network Open.*
